# Extracting Family History of Patients From Clinical Narratives: Exploring an End-to-End Solution With Deep Learning Models

**DOI:** 10.2196/22982

**Published:** 2020-12-15

**Authors:** Xi Yang, Hansi Zhang, Xing He, Jiang Bian, Yonghui Wu

**Affiliations:** 1 Department of Health Outcomes and Biomedical Informatics College of Medicine University of Florida Gainesville, FL United States; 2 Cancer Informatics Shared Resource University of Florida Health Cancer Center Gainesville, FL United States

**Keywords:** family history, information extraction, natural language processing, deep learning

## Abstract

**Background:**

Patients’ family history (FH) is a critical risk factor associated with numerous diseases. However, FH information is not well captured in the structured database but often documented in clinical narratives. Natural language processing (NLP) is the key technology to extract patients’ FH from clinical narratives. In 2019, the National NLP Clinical Challenge (n2c2) organized shared tasks to solicit NLP methods for FH information extraction.

**Objective:**

This study presents our end-to-end FH extraction system developed during the 2019 n2c2 open shared task as well as the new transformer-based models that we developed after the challenge. We seek to develop a machine learning–based solution for FH information extraction without task-specific rules created by hand.

**Methods:**

We developed deep learning–based systems for FH concept extraction and relation identification. We explored deep learning models including long short-term memory-conditional random fields and bidirectional encoder representations from transformers (BERT) as well as developed ensemble models using a majority voting strategy. To further optimize performance, we systematically compared 3 different strategies to use BERT output representations for relation identification.

**Results:**

Our system was among the top-ranked systems (3 out of 21) in the challenge. Our best system achieved micro-averaged F1 scores of 0.7944 and 0.6544 for concept extraction and relation identification, respectively. After challenge, we further explored new transformer-based models and improved the performances of both subtasks to 0.8249 and 0.6775, respectively. For relation identification, our system achieved a performance comparable to the best system (0.6810) reported in the challenge.

**Conclusions:**

This study demonstrated the feasibility of utilizing deep learning methods to extract FH information from clinical narratives.

## Introduction

Patients’ family history (FH) is a critical risk factor associated with numerous diseases [1-3] such as diabetes [4], coronary heart disease [5], and multiple types of cancers [6-9]. For example, a previous study showed that if a female patient has both her mother and sister having breast cancer, her relative risk [10] of having breast cancer increased 3.6 times compared with people without such FH [11]. Knowing the FH of patients can greatly help the prevention, diagnosis, and treatment of various diseases. However, FH is not well structured in current electronic health record databases but often documented as free text in clinical notes. Manually extracting patients’ FH information is a labor-intensive and time-consuming procedure that cannot be scaled up. Natural language processing (NLP) is the key technology to build automated computational models to extract patients’ FH from clinical narratives in their electronic health records.

In the past 2 decades, researchers have invested a significant amount of effort into developing various methods and tools to extract patients’ information from clinical narratives [12-14]. The clinical NLP community has organized a series of shared tasks for retrieving various patients’ information from clinical narratives including diseases or disorders [15-17], adverse drug events [18,19], and medical temporal relations [20]. Both rule-based and machine learning–based methods have been examined, and clinical NLP systems such as MetaMap [21], cTAKES [22], and CLAMP [23] have been developed. More recently, deep learning–based approaches have demonstrated superior performances in many NLP tasks [24]. For example, the long short-term memory-conditional random fields (LSTM-CRFs) architecture [25], which is a modified implementation of the recurrent neural network, has been widely adopted for named entity recognition (NER) tasks in both general and clinical domains. Later, a newly emerged bidirectional encoder representations from transformers (BERT) model achieved state-of-the-art performances in 20 NLP benchmarks in the general English domain [26] and demonstrated promising results in several clinical NLP tasks [27-29]. However, there are only a handful of studies focused on extracting FH of patients [30-32], which is more complicated than merely extracting information of the patients as it relates to various family members of the patient. FH often contains information from different aspects of the patients, including family members, their living status, and their diseases or disorders. Furthermore, patient’s family members need to be characterized by family role (eg, mother) and family side (eg, maternal). Besides, there are limited clinical corpora annotated for FH. The 2018 BioCreative/OHNLP Challenge [33,34] is the first shared task focusing on FH extraction. During that challenge, Shi et al [35] explored a joint deep learning approach and achieved the best performance among all participated teams. In 2019, the National NLP Clinical Challenge (n2c2) organized shared tasks to solicit advanced NLP methods for extracting FH information from clinical text. The 2019 n2c2 open shared task consisted of 2 subtasks: (1) NER for family members and observations (ie, diseases or disorders); and (2) identifying relations between family members, observations, and living status. Participants were required to identify mentions of FH and present a family member as a combination of family role (eg, mother) and family side (eg, maternal) and living status as a score derived from the healthy and alive state.

This paper presents our end-to-end FH extraction system developed during the 2019 n2c2 open shared task as well as new transformer models we developed after the challenge. During this challenge, we adopted an LSTM-CRF model for NER and a BERT-based model for relation identification. Our best submission was ranked fifth in subtask 1 and third in subtask 2. After the challenge, we further explored a BERT-based model for NER and demonstrated better performances in both subtasks.

## Methods

### Data

This study used the data set developed by the 2019 n2c2 open shared task organizers consisting of 216 clinical notes extracted from the Mayo Clinic data warehouse. The organizers split the corpus into a training set of 99 notes and a test set of 117 notes. Three types of concepts were annotated, including family members, observations (ie, diseases and disorders), and living status. There are also 2 types of relations annotated among family members, observations, and living status. The organizers provided annotations at (1) entity level (ie, the words and phrases about FH), and (2) document level, where the multiple mentions of the same FH were aggregated. Table 1 shows the descriptive statistics of the corpus.

**Table 1:** Descriptive statistics of the challenge data set.

Corpus information, annotation type, and annotation category				2019 n2c2 family history challenge corpus		
				Training set		Test set
Number of notes				99		117
**Entity-level annotation**						
	**Concept**					
		Family members	803		N/A	
		Observations	978		N/A	
		Living status	415		N/A	
**Document-level annotation**						
	**Concept**					
		Family members	667		638	
		Observations	930		983	
	**Relation**					
		Family members—observations	740		755	
		Family members—living status	376		349	

### The Family History Extraction System

**Figure 1:**
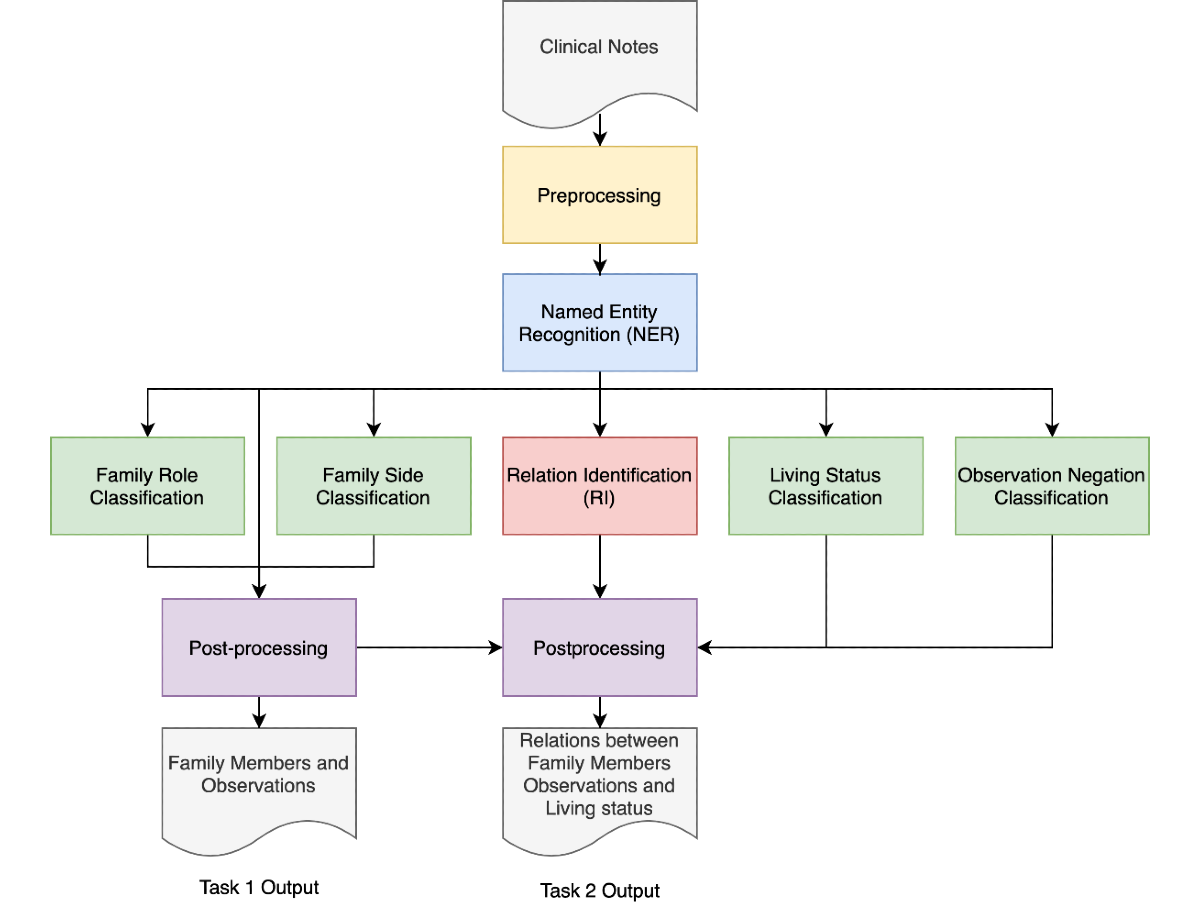
Overview of our family history extraction system.

Figure 1 shows the system architecture for our end-to-end FH extraction system. Our system has 5 modules including preprocessing, NER, classification, relation identification, and postprocessing. The preprocessing module contains standard NLP procedures including tokenization, sentence boundary detection, and data format transformation. In the NER module, we explored state-of-the-art NLP models, including LSTM-CRFs and BERT to identify FH concepts. The relation identification module applied deep learning models to determine the relations among FH concepts. The postprocessing module aggregated the entity-level results to the document level for both concept extraction and relation identification subtasks.

### Extracting Family History Concepts

The concept extraction subtask focused on detecting the mentions of family members and observations. We approached this subtask as a typical NER problem and applied deep learning–based models. Following the standard machine learning–based NER procedure, we converted the annotations using the beginning-inside-outside (BIO) tagging scheme [36,37], where “B” indicates the first token of a concept, “I” indicates tokens inside of a concept, and “O” indicates tokens that do not belong to any concepts. Thus, we converted information extraction problem into a sequence labeling task to assign each word with one of the predefined NER labels (“B,” “I,” or “O”). We explored 2 deep learning–based models including LSTM-CRFs and BERT.

**Figure 2:**
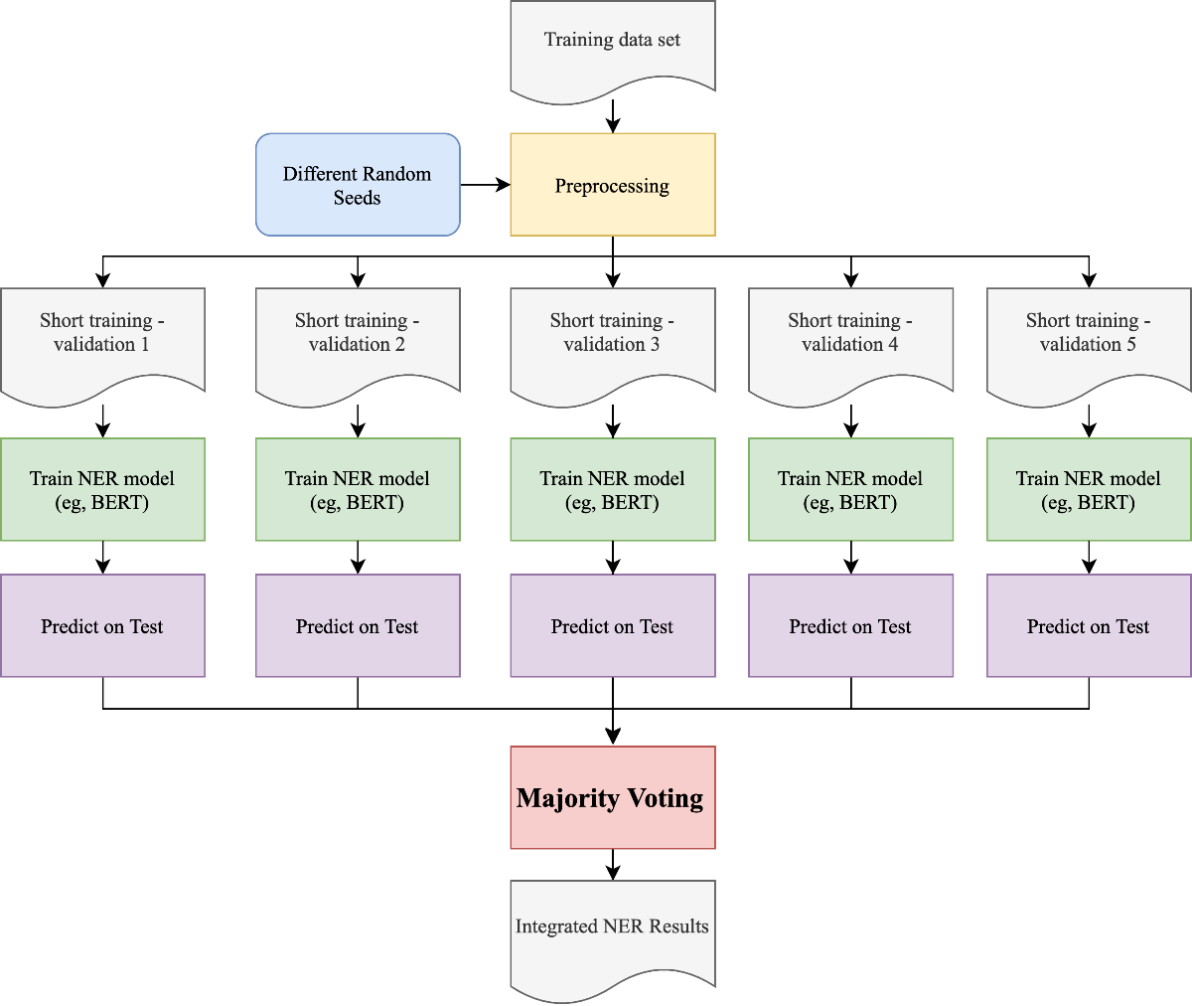
The majority voting strategy to ensemble NER models. BERT: bidirectional encoder representations from transformers; NER: named entity recognition.

Previous studies [38-41] have shown that adopting an ensemble method could further improve the clinical NER performances. Thus, we adopted the majority voting strategy to integrate the different NER models as shown in Figure 2. More specifically, we randomly (based on a random seed) split the training data into a short training data and a validation data at a 9:1 ratio. We trained deep learning models using the short training data and selected the best checkpoints based on the model performance on the validation data. By repeating the procedure 5 times with different random seeds, we obtained 5 different models. In each training procedure, we used different short training data and validate data but the same hyperparameters (ie, the optimized hyperparameters used for training the single BERT NER model). Then, the majority voting strategy was used to vote among the 5 models. Here, we use a suffix “-EN” to indicate the ensemble method. For example, we used “LSTM-CRFs-EN” to denote the ensemble model of LSTM-CRFs, and “BERT-EN” to denote the ensemble model of using BERT.

### Determining Family Role and Family Side

This task is to determine the family role and family side for the mentions of FH. There are a total number of 15 types of family roles defined in this challenge, including father, mother, sister, parent, brother, grandmother, grandfather, grandparent, daughter, son, child, cousin, sibling, aunt, and uncle. There are 3 predefined family sides including maternal, paternal, and not applicable. We approached the 2 tasks as classification problems. Previous studies [35,42] approached the 2 tasks using rule-based methods; here, we applied deep learning–based classification methods as machine learning–based methods have shown a better generalizability.

### Relation Identification

Typically, relation identification consists of 2 steps: (1) determine whether there is a relation between 2 entities; and (2) classify the correct relation type. In this study, we formulated the relation identification as a binary classification problem. We presented each relation as a pair of 2 entities and used contextual information around the entities to classify these pairs into categories as “in-relation” or “nonrelation” (no relation between entities). Then, we further categorized the “in-relation” entity pairs into either “family member—living status” group or “family member—observation” group based on the entity types: if 1 of the entities in an entity pair is observation, we classify it as “family member—observation”; if one of the entities in an entity pair is living status, we classify it as “family member—living status.”

#### Candidate Concept Pairs Generation

Theoretically, there might be relations between any pair of FH concepts. Thus, a naïve way is to generate candidate pairs from all combinations of clinical concepts in document level. However, a previous study [43] has reported that this method often generates too many negative samples (ie, nonrelation), causing an extremely imbalanced positive-to-negative sample ratio. To alleviate this issue, we applied the following heuristic rule to reduce the combinations: only keep the concept pairs composed of a family member entity as the first element and a nonfamily member entity as the second element. We also looked into the cross-distance of pairs—defined as the number of sentence boundaries between the 2 entities (eg, 0 for single-sentence relations, and 1 for relations across 2 sentences). In the training set, the cross-distance ranges from 0 to 10 and we found that 96% of the annotated relations have cross-distances less than 3. Therefore, we only consider candidate pairs with cross-distances less than 3. Previous studies [44,45] developed individual classifiers to handle relations with different cross-distance; here, we developed a unified BERT-based classifier to handle all candidate pairs with various cross-distances as the BERT model is able to learn both token- and sentence-level representations.

#### Handling Negations

In this study, we approached negation detection as a binary classification problem—classify the observation entity into 2 predefined categories including “negated” and “non-negated.” We developed a BERT-based classifier for negation detection. In our system, we performed the negation detection for each observation entity and then integrated the results into relations. We only used the negation annotations from the challenge data set and did not use any external resources.

#### Assessing the Living Status Scores

For the relations between “family member—living status,” the participants were required to assess the living status using scores of 0, 2, or 4, where 0 indicates not alive, 2 indicates alive but not healthy, and 4 indicates alive and healthy. We approached this task as a classification task—to categorize a living status entity into one of 3 score categories (ie, 0, 2, and 4). We developed a BERT-based classifier to classify each living status entity into a category according to its context.

### Deep Learning Models

#### LSTM-CRFs

In this study, we adopted an LSTM-CRFs architecture proposed by Lample et al [25]. The model has 2 bidirectional LSTM layers: one for learning representations at the character level and the other for learning those at the word level. The model utilizes a CRFs layer to decode the LSTM hidden states to BIO tags. We screened 4 different word embeddings following a similar procedure reported in our previous study [46] and found that the Common Crawl embeddings—released by Facebook and trained using the fastText on the Common Crawl data set [47]—achieved better performance compared to other embeddings on a validation data set. Thus, we used the Common Crawl embeddings for all LSTM-CRFs models.

#### BERT

The BERT model is a multilayer transformer encoder model implemented using the self-attention mechanism [48], which is pretrained by combining the masked language modeling method and the next sentence prediction task. BERT has 2 versions featuring different model sizes, including a BASE version with 12 transformer layers and 110 million parameters, and a LARGE version with 24 transformer layers and 340 million parameters [26]. There are 2 steps to apply BERT for various downstream NLP, including (1) pretraining a BERT model using large unlabeled corpora and (2) fine-tuning the pretrained model using task-specific annotated corpora. In this study, we adopted the general pretrained BERT-LARGE model and fine-tuned it individually for each subtask (ie, concept extraction and relation identification) using the annotated data set developed in this challenge. We denoted the BERT-based NER model as BERT-*ner*, and the BERT-based family member attributes (ie, family role, side of family, negation, living status) classification module as BERT-*cls* and relation extraction module as BERT-*rel*.

**Figure 3:**
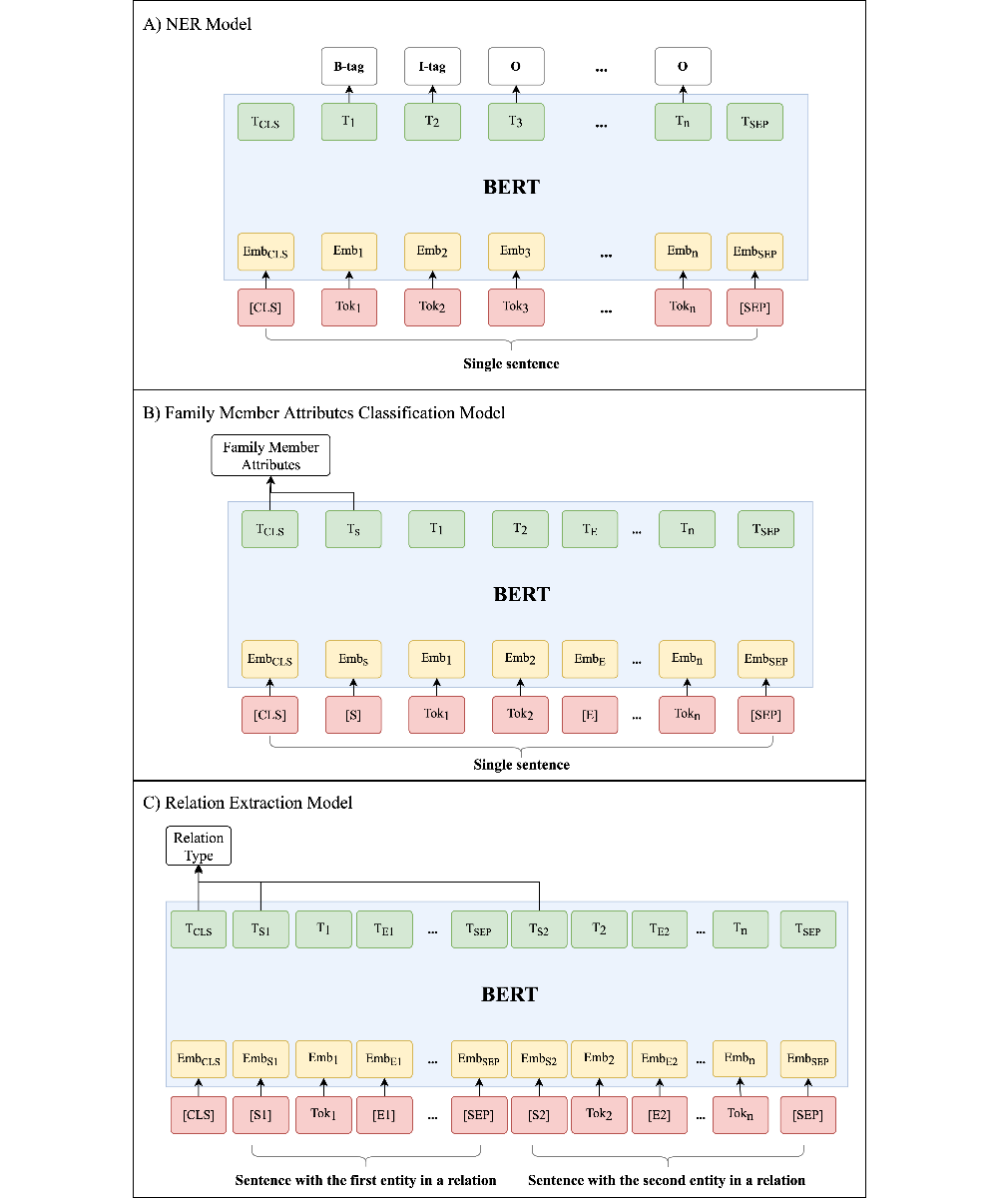
Illustration of BERT models for (A) NER, (B) family member attributes (including side and role of family members, negation of observations, and living scores) classification, and (C) relation extraction. BERT: bidirectional encoder representations from transformers; NER: named entity recognition.

Figure 3 illustrates the fine-tuning procedure for BERT. For token Tok_i_, its input embedding and contextual representation are denoted as Emb_i_ and T_i_. The [CLS] and [SEP] are 2 special symbols designed to format the input sequences. In this study, we also introduced a pair of entity marker including [S] and [E] to differentiate the target entity from other entities in the same sentence, where [S] indicates the start position and [E] indicates the end position. For NER (Figure 3A), the input for BERT model is a sequence of tokens, and the output is a sequence of distributed representation. Then, we used a linear layer to calculate a score for each BIO tag. Based on the entities, we developed classifiers to determine related attributes (Figure 3B). To distinguish between the target entity and other entities in the same sentence, we inserted entity markers (ie, [S] and [E]) in front of and after the target entity. For example, the input sequenced in Figure 3B contains the target entity (ie, Tok_1_ and Tok_2_) surrounded by the entity markers and other entities (eg, Tok_n_). Then, we concatenated the representations corresponding to the [CLS] and [S] tokens and calculated a score for each predefined class label using a linear layer. For relation identification (Figure 3C), we determined the relation type based on the contextual information of 2 concepts in a relation. Therefore, the input consisted of 2 sentences linked by the special token [SEP], where each sentence contains 1 of the 2 entities in the relation. We used 2 sets of entity markers (ie, [S1], [E1], and [S2], [E2]) to label the entities. If the 2 entities of a relation are in the same sentence, then the 2 model-input sentences are the same but with different entity markers. To determine the relation category, we concatenated the representations from [CLS] and 2 start position entity makers ([S1] and [S2]) and used a linear layer to calculate a score for each predefined relation type.

### Experiments and Evaluations

In this study, we reused the LSTM-CRFs model developed in our previous study [49] and implemented the BERT-based models on top of the Transformers library [50] implemented in PyTorch [51]. We used the following parameters to initialize the LSTM-CRFs: the character embedding dimension was 25, the word embedding dimension was 100, the character-level bidirectional LSTM layer dimension was 25, the word-level bidirectional LSTM layer was 100 with a dropout probability of 0.5, the learning rate was fixed at 0.005, and the stochastic gradient descending applied a gradient clapping at [–5.0, 5.0]. The character embeddings were randomly initialized and the word embeddings were initiated using embeddings from fastText [47] (ie, containing 2 million word vectors trained on Common Crawl). We initialized all BERT-based models using the BERT-LARGE pretrained on the general English corpus and fine-tuned them with the default model parameter settings. To train NER models, we randomly (using random seeds for reproducibility) split the original training set (99 notes) into a short training set of 89 notes and a development set of 10 notes. The best NER models were selected according to the performance on the development set. We optimized 2 hyperparameters, including the number of epochs and batch size, via fivefold cross-validation. Table 2 summarizes the optimized hyperparameters. We conducted all experiments using 2 NVIDIA P6000 graphics processing units (GPUs). We used the official evaluation script provided by the 2019 n2c2 open shared task organizers to calculate the evaluation scores on the test set. Evaluation metrics as micro-averaged precision, recall, and F1 score were used for both subtask 1 and subtask 2.

**Table 2:** The optimized hyperparameters of BERT-based models for various tasks.

Task	Pretrained model	Number of epochs	Batch size	Learning rate
NER^a^	BERT^b^-LARGE	30	4	1.00 × 10^–05^
Negation classification	BERT-LARGE	5	8	1.00 × 10^–05^
Side of family classification	BERT-LARGE	10	4	1.00 × 10^–05^
Role of family classification	BERT-LARGE	5	8	1.00 × 10^–05^
Living status classification	BERT-LARGE	6	8	1.00 × 10^–05^
Relation identification	BERT-LARGE	12	16	2.00 × 10^–05^

^a^NER: named entity recognition.

^b^BERT: bidirectional encoder representations from transformers.

## Results

Table 3 compares our 4 systems for conception extraction and relation identification. Our best submission during the original challenge (LSTM-CRFs-EN + BERT-*cls* +BERT-*rel*) achieved F1 scores of 0.7944 and 0.6544 for subtask 1 and subtask 2, respectively, which is the third best system of this challenge among 17 participants. After the challenge, we further explored the BERT model for NER and the combination of BERT-*ner*-EN, BERT-*cls*, and BERT*-rel* achieved better F1 scores of 0.8249 and 0.6775 for the 2 subtasks, respectively. Compared to our best system developed during the challenge (LSTM-CRFs-EN + BERT-*cls* + BERT*-rel*), the new system (BERT*-ner-*EN + BERT-*cls* + BERT*-rel*) improved the F1 scores by 0.0305 and 0.0235 for the 2 subtasks, respectively. Our best relation identification performance was comparable to the best result reported in this challenge (0.6775 from us versus 0.6810 reported in this challenge).

**Table 3:** The micro-average performances for concept extraction and relation identification.a

Models	Subtask 1 (concept extraction)			Subtask 2 (relation identification)				
	Precision	Recall	F1 score		Precision	Recall	F1 score	
LSTM^a^-CRFs^b^ + BERT^c^-*cls* + BERT-*rel*	0.7760	0.8087	0.7920		0.7343	0.5465	0.6266	
LSTM-CRFs-EN + BERT-*cls* + BERT-*rel*^d^	0.7969	0.7920	0.7944		0.6995	0.6184	0.6544	
BERT-*ner* + BERT-*cls* + BERT-*rel*	0.8060	0.8105	0.8083		0.7140	0.6252^e^	0.6667	
BERT-*ner*-EN + BERT-*cls* + BERT-*rel*	0.8301^e^	0.8198^e^	0.8249^e^		0.7421^e^	0.6233	0.6775^e^	

^a^LSTM: long short-term memory.

^b^CRFs: conditional random fields.

^c^BERT: bidirectional encoder representations from transformers.

^d^Our best system developed during the challenge.

^e^The best performances.

Table 4 compares the detailed performance of LSTM-CRFs and BERT-*ner* for FH extraction. Compared with LSTM-CRFs, the BERT-*ner* model achieved a remarkably higher F1 score for the observation concepts (0.8094 for BERT-*ner* versus 0.7833 for LSTM-CRFs), but marginally lower performance for the family member concepts (0.8066 for BERT-*ner* versus 0.8069 for LSTM-CRFs). Table 4 also demonstrated that our ensemble strategy improved the performance of FH extraction. For example, the BERT-*ner*-EN, which was ensembled from 5 different BERT-*ner* models, outperformed the single BERT-*ner* model by about 2% for family members and about 1.5% for observations.

**Table 4:** A comparison of LSTM-CRFs and BERT for subtask 1 (concept extraction).

Model and concept		Precision	Recall	F1 score
**LSTM-CRFs^a,b^**				
	Family member	0.8480	0.7686	0.8069
	Observation	0.7382	0.8342	0.7833
**LSTM-CRFs-EN**				
	Family member	0.8451	0.7868	0.8149
	Observation	0.7685	0.7953	0.7817
**BERT^c^-*ner***				
	Family member	0.8059	0.8072	0.8066
	Observation	0.8061	0.8127	0.8094
**BERT-*ner*-EN**				
	Family member	0.8294	0.8229	0.8261
	Observation	0.8306	0.8178	0.8241

^a^LSTM: long short-term memory.

^b^CRFs: conditional random fields.

^c^BERT: bidirectional encoder representations from transformers.

Table 5 compares the performance of relation identification for each relation category. Similar to the concept extraction results, the BERT-*ner*-EN + BERT-*cls* + BERT-*rel* system achieved the best F1 scores of 0.6821 and 0.6760 for the “family member—living status” and “family member—observation” relations, respectively. Compared to the LSTM-CRFs, the BERT-*ner*–based systems achieved better recalls.

**Table 5:** The category-level performances for subtask 2 (relation identification).

Model and relation		Precision		Recall		F1	
**LSTM-CRFs^a,b^ + BERT^c^-*cls* + BERT-*rel***							
	Family member—living status		0.7039		0.6132		0.6554
	Family member—observation		0.7452		0.5269		0.6174
**LSTM-CRFs-EN + BERT-*cls* + BERT-*rel***							
	Family member—living status		0.6773		0.6676		0.6724
	Family member—observation		0.7071		0.5993		0.6487
**BERT-*ner* + BERT-*cls* + BERT-*rel***							
	Family member—living status		0.6583		0.6734		0.6657
	Family member—observation		0.7341		0.6111		0.6670
**BERT-*ner*-EN + BERT-*cls* + BERT-*rel***							
	Family member—living status		0.6912		0.6734		0.6821
	Family member—observation		0.7603		0.6086		0.6760

^a^LSTM: long short-term memory.

^b^CRFs: conditional random fields.

^c^BERT: bidirectional encoder representations from transformers.

## Discussion

### Overview

Patients’ FH is a critical risk factor associated with numerous diseases. Clinical NLP systems that automatically extract FH from clinical narrative are needed for many clinical studies and applications. The 2019 n2c2 organized shared tasks to assess current NLP methods for FH information extraction from clinical narratives. We participated in both subtasks and our system (LSTM-CRFs-EN + BERT-*cls* + BERT-*rel*) achieved the third best performance (F1 of 0.6544) among all the 21 submitted systems from 17 teams that participated in subtask 2. After the challenge, we further explored the BERT models for the concept extraction and improved our system in both concept extraction and relation identification.

### Principal Findings

We observed that the BERT-*ner* model achieved both better precision (0.8060 versus 0.7760) and recall (0.8105 versus 0.8087) for clinical concept extraction compared to the LSTM-CRFs, which is consistent with a recent study by Si et al [52]. We also noticed that the single BERT-*ner* mode even achieved a higher F1 score of 0.8083 than the ensembled LSTM-CRFs model (LSTM-CRFs-EN with F1 score of 0.7944). Ensemble is an effective strategy to further improve the performance of NER. For example, the ensembled BERT model (ie, BERT-*ner*-EN, which was ensembled from 5 individual BERT-*ner* models) improved the concept extraction performance to 0.8249, compared to the single BERT model (F1 score of 0.8083). The performance improvement of the ensembled model was mainly in precision, suggesting that the ensembled models may reduce the classification errors in NER. However, further studies should examine whether our observation is related to the size of training corpus (relatively small, only 99 notes).

Most of the previous studies applied rule-based solutions to determine the family roles and family sides [34]. In this study, we adopted a pure machine learning–based solution. The experimental results showed that the BERT-based classifiers were feasible to determine the family roles, family sides, negation of observations, and living status scores. Another advantage of our method is that machine learning–based models generally have a better generalizability than rule-based systems and are easy to scale up. FH information has many variations from one patient to another, which makes the development of rules time-consuming and expensive.

In our system, we only used the sentences containing the concepts to classify the family member attributes. We also examined a strategy to include both the proceeding and following sentences. However, the experimental results based on the fivefold cross-validation on the training set showed that adding the context information did not improve the performance. One potential reason may be that most of the key information for classifying the family member attributes is located in the same sentence where the concepts (ie, family member or observation) are located. Besides, there might be potential noises brought in when including the context sentences.

A previous study [53] examined various input encoding and output representation of using BERT for relation extraction, and concluded that using representations aggregated from the start position entity markers (eg, [S1] and [S2] in Figure 3C) was the best practice. In this study, we re-evaluated 3 types of BERT output representations, including (1) the representation of the [CLS] only, (2) the representations aggregated from the start position entity markers, and (3) the representations aggregated from the [CLS] and the start position entity markers. Our results showed that option (3) led to a remarkably higher averaged F1 score (0.8975) compared to the other 2 representations (0.8851 and 0.8904). A possible reason is that the representations captured in the special token [CLS] and the representations of the start position markers contain contextual information that is complement to each other. Further studies are needed to continue examining more efficient methods for encodings and representations.

This study has limitations. First, there are limited clinical corpora for FH-related information extraction as annotating clinical notes is expensive and time-consuming. A potential solution is to use data augmentation techniques such as generative adversarial networks, which have been applied for medical imaging data [54,55]. There are preliminary research works demonstrating that generative adversarial networks could be utilized to synthesize clinical text [56]. Second, our system is a 2-stage pipeline where the errors generated in the NER will be propagated to relation extraction. We will explore potential solutions such as joint learning algorithms to alleviate this issue in our future work.

### Error Analysis

Table 6 shows the confusion matrix generated for the concept extraction (subtask 1) based on our best NER model (ie, BERT-*ner*-EN). The confusion matrix showed that our system could efficiently identify family member entities. However, it is challenging for our system to differentiate the nonconcept terms for both family members and observations. For concept extraction, our system had relatively lower performances for “parent,” “grandparent,” “child,” and “siblings.” One possible reason is that the training set contains limited annotations of these entities. For example, the “parent” entity only appeared once and the “grandparent” entities appeared 6 times in the training data set. We also found that our system identified some observations not annotated in the test set. For example, in the sentence “The father also had a history of vascular surgery, a long history of smoking, and has had hip replacement,” our system extracted observations of “vascular surgery,” “smoking,” and “hip replacement,” which were annotated in the challenge corpus.

**Table 6:** The confusion matrix table for the NER (subtask 1).^a^

Entity type	Model prediction		
	FM^b^	OB^c^	NC^d^
FM	525	0	113
OB	0	799	178
NC	108	163	N/A^e^

^a^FM, OB, and NC are considered gold standard.

^b^FM: family members.

^c^OB: observations.

^d^NC: not a concept.

^e^N/A: not applicable.

### Conclusions

Extracting patients’ FH information from clinical narratives is a challenging NLP task. This study demonstrated the efficiency of deep learning–based NLP models for extraction of FH. Our system and pretrained models can be accessed at [57]. We believe our system could help other researchers to extract and leverage patient’s FH documented in clinical narratives in their studies.

## Abbreviations

BERT: bidirectional encoder representations from transformers
BIO: beginning-inside-outside
CRFs: conditional random fields
FH: family history
LSTM: long short-term memory
n2c2: National NLP Clinical Challenge
NER: named entity recognition
NLP: natural language processing
GPU: graphics processing unit
